# The art of oocyte meiotic arrest regulation

**DOI:** 10.1186/s12958-018-0445-8

**Published:** 2019-01-05

**Authors:** Bo Pan, Julang Li

**Affiliations:** Department of Animal Biosciences, University of Guelph, 50 Stone Road E, Building #70, Guelph, ON N1G 2W1 Canada

**Keywords:** Oocyte, Granulosa cell, Meiotic arrest, Meiotic assumption, cGMP, cAMP

## Abstract

A central dogma of mammalian reproductive biology is that the size of the primordial follicle pool represents reproductive capacity in females. The assembly of the primordial follicle starts after the primordial germ cells (PGCs)-derived oocyte releases from the synchronously dividing germline cysts. PGCs initiate meiosis during fetal development. However, after synapsis and recombination of homologous chromosomes, they arrest at the diplotene stage of the first meiotic prophase (MI). The diplotene-arrested oocyte, together with the surrounding of a single layer of flattened granulosa cells, forms a basic unit of the ovary, the primordial follicle. At the start of each estrous (animal) or menstrual cycle (human), in response to a surge of luteinizing hormone (LH) from the pituitary gland, a limited number of primordial follicles are triggered to develop into primary follicles, preantral follicles, antral follicles and reach to preovulatory follicle stage. During the transition from the preantral to antral stages, the enclosed oocyte gradually acquires the capacity to resume meiosis. Meiotic resumption from the prophase of MI is morphologically characterized by the dissolution of the oocyte nuclear envelope, which is generally termed the “germinal vesicle breakdown” (GVBD). Following GVBD and completion of MI, the oocyte enters meiosis II without an obvious S-phase and arrests at metaphase phase II (MII) until fertilization. The underlying mechanism of meiotic arrest has been widely explored in numerous studies. Many studies indicated that two cellular second messengers, cyclic adenosine monophosphate (cAMP) and cyclic guanosine monophosphate (cGMP) play an essential role in maintaining oocyte meiotic arrest. This review will discuss how these two cyclic nucleotides regulate oocyte maturation by blocking or initiating meiotic processes, and to provide an insight in future research.

## Intra-oocyte elevated cAMP maintains meiotic arrest

**Fig. 1:**
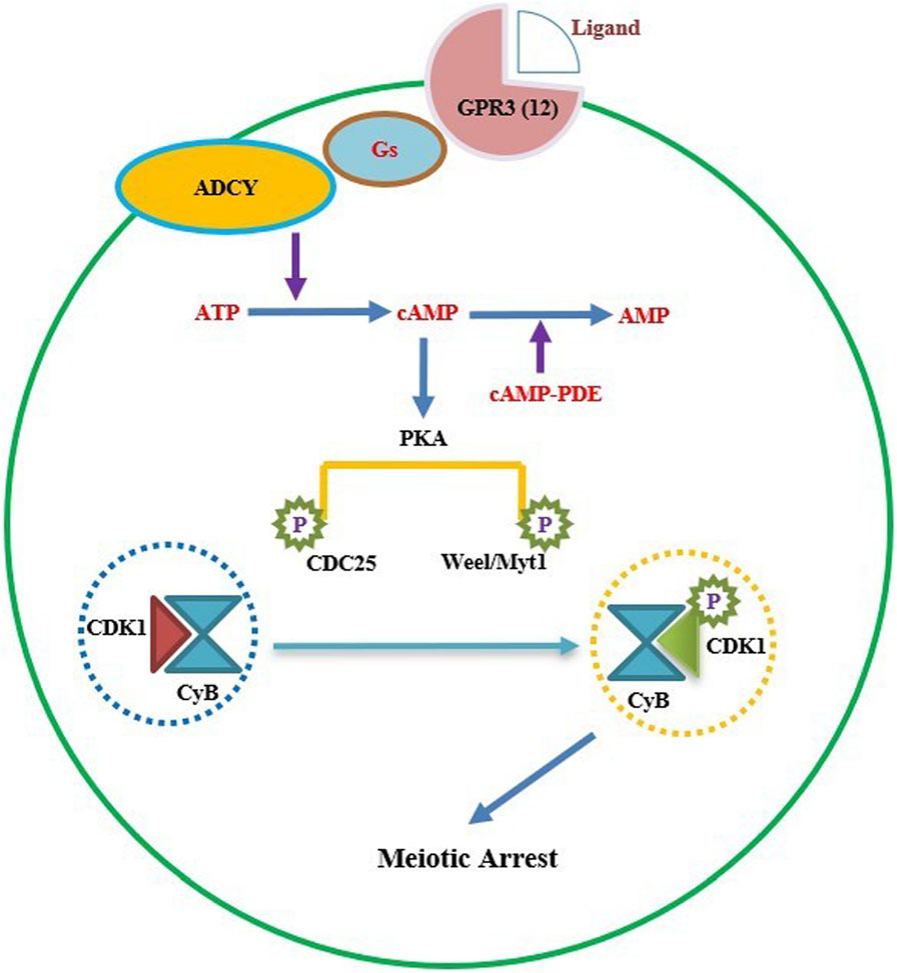
Schematic depiction of coordination between GPR-Gs-ADCY and cAMP-PDE maintain high level of cAMP in the oocyte. After the trans-membrane receptor GPR2 is activated, its conformation changes. GPR2 couples with Gs protein and initiates Gs-adenylyl cyclise to convert ATP to cAMP. This process is accompanied by the inhibition of cAMP-PDE from unknown signalling preventing the hydrolysis of cAMP, resulting the accumulation of cAMP in the oocyte. The increase of cAMP in the oocyte activates the PKA, causing the phosphorylation of CDC25B and Weel/Myt1, which in return inactivate MPF, ultimately inducing meiotic arrest at the diplotene stage

In a wide variety of animal species, a universal cytoplasmic maturation-promoting factor [also termed as maturation [M-phase] promoting factor, MPF] plays a pivotal role in GVBD and the subsequent maturational events in the oocyte [1]. MPF is a heterodimer composed of Cyclin-dependent kinase 1 (CDK1; a catalytic subunit; also termed p34^cdc2^) and Cyclin B (B1, B2 and B3, a regulatory subunit). CDK1 phosphorylates specific serine and threonine residues of its target proteins, but itself is not sufficient for kinase activity, thus it is necessary to bind with the Cyclin B which ensures CDK1 functions with the appropriate substrate [1–3]. In the oocyte, it is well documented that the elevated intracellular cAMP level continuously activates protein kinase A (PKA), which subsequently results in the phosphorylation and activation of nuclear kinase Weel/MytI. This activation in turn inactivates the cell division cycle 25B (CDC25B), which is the activator of cyclin-dependent kinase, thus ultimately maintaining the MPF in an inactive state (so-called inactive MPF) by inhibiting the phosphorylation of CDK1 at Thr14 and Tyr15 [1, 4–6] (Fig. 1). The cell division cycle 25A (CDC25A) also was suggested to play a role in the resumption of meiosis as exogenous CDC25A overcame cAMP-mediated maintenance of meiotic arrest [7]. However, its role in oocyte maturation is not clear because CDC25A deficient mice are embryonic lethal [8]. In contrast, CDC25B deficient female mice are sterile due to permanent meiotic arrest resulting from low MPF activity. In addition, unlike CDC25B which localizes to the cytoplasm of GV oocytes and translocates to the nucleus shortly before GVBD occurs, CDC25A is exclusively localized to the nucleus prior to GVBD [7]. Thus, investigation of CDC25A’s function in the resumption of meiosis represents an interest for the future research.

It has been believed that antral follicular somatic cells including the theca cells, the mural granulosa cells (MGC) and the cumulus granulosa cells (CGC), produce cAMP and consecutively transfer into the oocyte thus maintaining MPF as inactive. This inactivation results in the fully-grown oocyte stop at the diplotene stage until the surge of pituitary-derived LH stimulation [9–11]. In supporting this notion, it was shown that, hormones or the adenylyl cyclase (ADCY) activator forskolin, increased cAMP in the oocyte in a cumulus cell-dependent manner in mice [12–14], rat [15], rabbit [16], and cow [17]. In addition, the elevated cAMP in MGCs by follicle-stimulating hormone (FSH) stimulation also increase permeability in gap junctions and changed in the cellular distribution of connexin43 (Cx43) [18], which is believed to deliver inhibitory signals cAMP from the follicular somatic cells to the oocyte to sustain the threshold level of intracellular cAMP level [19, 20].

However, this long-standing hypothesis has been challenged by the finding of Gs-GPR-ADCY cascade in the oocyte. It is well known that the Gs protein couples with receptors to ADCY and is required for hormone-stimulated cAMP generation in vast kinds of tissues [21]. Study showed injection of inhibitory Gs antibody or a dominant negative form of Gs into the follicle-enclosed mouse oocyte resulted in meiosis resumption [22, 23]. Given that the Gs protein by itself has no detectable constitutive activity, a Gs protein–coupled receptor 3 (GPR3) was identified in the oocyte membrane and required to keep Gs active to sustain a basal level of cAMP to maintain mouse oocyte meiotic arrest [24]. In direct evidence generated by GPR3 deficient mouse model, it was found that GPR3 knockout mouse oocytes showed spontaneous meiotic resumption at early antral stage; in contrast, this phenotype was blocked by the injection of *GPR3* RNA back into the oocyte [24, 25]. Similarly, GPR3 was consistently detected in the pig oocyte through GV to MII stage, and the injection of specific small interfering double-stranded RNA (siRNA) for GPR3 stimulated meiotic resumption of oocytes; in contrast, the over-expression of GPR3 by the injection of GPR3 mRNA back into the oocyte significantly inhibited meiotic resumption [26]. This is consistent with the results observed in the *Xenopus* oocyte that up-regulation of GPR3 increased intraoocyte cAMP leading to suppression of meiosis resumption [27]. However, in the Atlantic croaker, cyprinid fish and zebrafish, another GPR superfamily member, orphan G protein Homology of GRP3 was identified and found to be involved in maintaining oocyte meiotic arrest [28]. Like GPR3, another GPR family member, GPR12 was detected in the oocyte of *Xenopus laevis*, mouse and rat [29]. Over-expressed GPR12 in *Xenopus laevis* oocytes prevented meiotic resumption induced by progesterone [29]. However, unlike the ablation of the GPR3 leads to mice oocyte meiotic arrest [30], GPR12-deficient mice showed no signs of precocious maturation, suggesting constitutive activity of GPR12 is not sufficient to maintain meiotic arrest by itself in the mice [29, 30].

In the oocyte, ADCY is the effector enzyme after the Gs protein causes the constitutive activation of Gs-coupled receptor. ADCY is part of a family of enzymes responsible for catalyzing the cyclization of adenosine triphosphate (ATP) into cAMP, thus maintains an elevated cAMP level within the oocyte [25]. Nine closely related transmembrane-bound (ADCY 1–9) genes showing significant sequence homology and sharing the same overall structure have been found in the human genome [31]. One member of ADCY family, ADCY3 was detected in both mouse and rat oocytes. In contrast, ADCY3 mRNA and protein expression are present at low level or barely detectable in either mural granulosa cell or cumulus granulosa cell, respectively [32]. In addition, ADCY3 deficient mouse oocyte showed defect in meiotic arrest in vivo and accelerated spontaneous maturation in vitro [32]. These observations are consistent with the studies that meiotic cell division in amphibian oocytes occurred after the inhibition of ADCY activity [33, 34]. However, the ADCY3-null mice neither completely abolishes the meiotic arrest in vivo nor cause hundred percentage of spontaneous maturation in vitro, possibly due to the compensation by some other ADCY members: ADCY1 and ADCY9. Unlike ADCY3, which is the only ADCY isoform identified in rats, these two isoforms were detected in mice oocyte as well [32]. In addition to the nine classic transmembrane-bound ADCY, a soluble adenylyl cyclase (sADCY) was recently identified as a widely expressed intracellular source of cAMP in mammal [35]; Unlike the rest of ADCYs that are membrane-bound and regulated by calcium/calmodulin concentration, sADCY is distributed throughout the cytoplasm and in cellular organelles, and is uniquely regulated by bicarbonate anions [36, 37]. Notably, the roles of sADCY in the male germ cell have been widely studied, however, no report in the oocyte has yet to be published [38, 39], thus more study regarding whether it is involved in the follicular events may represent another big interest for the reproductive research.

It’s known that along with the GPR-Gs-ADCY signalling cascade generated intrinsic cAMP, an important enzyme cAMP-Phosphodiesterase (cAMP-PDE) is also required to be inactivated to prevent the degradation of cAMP, to maintain an elevated cAMP level sufficient for meiosis arrest within the oocyte. The main function of cAMP-PDE is to dephosphorylate cAMP into AMP causing a reduction in the cAMP levels. Eleven distinct phosphodiesterase isoenzymes (PDE1–11) which are encoded by at least 20 gene types have been found in mammal. They are differentially expressed and regulated in different cellular and tissue locations despite having a similar structure [40, 41]. In contrast to PDE4D and PDE4B uniquely locate to the mural granulosa cells, cumulus granulosa cell and theca cell; PDE3 is specifically localized within the rat oocyte [42–44]. In addition, PDE3 has been widely investigated and shown a prominent expression in the oocyte but not the somatic compartment of mice [45], bovine [46] and pig [47]. Treatment with the specific PDE3 inhibitor, Cilostamide, during in vitro cumulus-oocyte-complex (COC) or denuded oocytes (DO) culture prevented COC spontaneous meiotic maturation and elevated intraoocyte cAMP level in mouse, bovine and human [48–50]. These results are consistent with that PDE3 null mouse oocyte which completely blocked meiosis either in vitro or in vivo studies [51]. However, given the function of oocyte-specific GPR3 is required for the maintenance of meiotic arrest in mice oocytes and consequently GPR3 null mice are infertile [52], the attempt of crossing of PDE3 null mice with GPR3 null mice resulted in partial recovery of female fertility [51]. These observations in the PDE3/GPR3 null mice strongly implicate a collaborated effect between PDE activity and GPR-Gs-ADCY signalling within the oocyte in the maintenance of meiosis arrest through sustaining a sufficient cAMP level. It seems that the oocyte develops a fine-tuning mechanism in the controlling meiotic arrest by integrating two pathways: [1] The activity of GPR3-Gs-ADCY cascade produces cAMP and thus continuously contributes to the maintenance of elevated intracellular cAMP level; [2] The inhibition of PED3 activity prevents the degradation of cAMP in the oocyte. The coordination of these two pathways sustains the oocyte arresting at the diplotene stage of meiosis I until LH surge initiates the meiotic resumption (Fig. 1).

It’s of a great interest to explore the mechanism of activating GPR3-Gs-ADCY signalling. The activated GPR3-Gs-ADCY signalling is initiated by the binding of GPR3 and its ligand(s). Given that GPR represents the largest family of cell-surface transmembrane proteins which can be activated by a wide variety of ligands, including ions, peptides, hormones, growth factors [53, 54], numerous studies have investigated the potential ligands for GPR3 in the follicle. For example, Lysophospholipids (LPs), a novel group of extracellular ligands for GPCRs is starting to draw researches’ attention (for more details, please refer to the review [55]). Three main well-studied LPs are lysophosphatidic acid (LPA), lysophosphatidylcholine (LPC), sphingosylphosphorylcholine (SPC), and sphingosine 1-phosphate (S1P). It was found treatment with the GPR3/12 ligands, SPC and S1P during incubation of mouse oocytes delayed spontaneous oocyte maturation [55–57]; these results are consistent with the studies that the expression of GPR3 and/or GPR12 is essential for maintaining meiotic arrest in mouse and rat oocytes [24, 30]. However, it’s notable that both S1P3 and GPR3 are detected in both mouse oocytes and cumulus granulosa cells, so the possibility that the initiation of GPR3-Gs-ADCY cascade is trigged by cumulus cell-derived ligands requires further investigation [55].

## Bidirectional communication between oocyte and follicular somatic cells

Once the oocyte is separated from the antral follicle and cultured within supportive media, the presence of spontaneous meiotic resumption has been observed in mammalian species, which suggests the follicular somatic cell, especially mural granulosa cells and cumulus granulosa cells, play an essential role in holding oocyte meiosis arrest in the antral follicle [58]. However, given the conclusion derived in the former section that the oocyte intrinsic cAMP is sufficient to maintain oocyte in meiotic arrest in vivo and in vitro, it seems paradoxical that the apparent function of follicular granulosa cell in holding oocyte meiosis arrest. Until recently, the finding that the suppression of the PDE3A activity in the oocyte caused by the diffusion of cGMP from the follicular granulosa cell to the oocyte which partly solves this paradox.

Like cAMP, cGMP is a water soluble second messenger found in eukaryotic and prokaryotic cells, despite of its role has long been overshadowed by that of cAMP. cGMP is produced from GTP under the activity of guanylyl cyclises; On the opposite direction, meanwhile inside of the cell, the cGMP-specific phosphodiesterase (cGMP-PDE) rapidly and continuously catabolizes the cGMP into to guanosine 5′ monophosphate (5′GMP) [59]. To date, it’s well accepted that the cGMP is synthesized by guanylyl cyclises in the mural granulosa cells and the cumulus granulosa cells and diffuses to the oocyte to inhibit the hydrolysis activity of PDE3A on cAMP, ultimately maintains the oocyte meiotic arrest at diplotene stage of prophase I in mice and pig [60, 61].

The main guanylyl cyclises in the mammalian follicle are natriuretic peptide receptors (NPR) [62]. There are three natriuretic peptide receptors (NPR-A [NPR1], NPR-B [NPR2], and NPR-C [NPR3]). NPR-A and NPR-B are guanylyl cyclase receptors, whereas NPR-C is non-guanylyl cyclase receptor and it is believed act as clearance or silent receptor [63]. Activation of NPR begins with interacting with its cognate receptors present on the plasma membrane, the natriuretic peptides (NPPs, also named ANPs), Natriuretic peptides comprise a family of three polypeptide hormones termed atrial natriuretic peptide (ANP), brain natriuretic peptide (BNP), and C-type natriuretic peptide (CNP). The NPRs and NPPs collaboratively form the natriuretic NPP/NPR signalling system and plays an important role in the regulation of cGMP synthesis within the ovary.

The selective expression of NPP and NPR among the mural granulosa cell, the cumulus granulosa cell, and the oocyte is believed to act as an important step in the regulation of the oocyte meiotic arrest (Tables 1, 2). Among the NPR family, NPR2 is the main trans-membrane NPR family member and is predominantly present in the cumulus granulosa cells, whereas, its cognate ligand NPPC, *NPPC* transcription was only expressed in the mural granulosa cells of mouse, human, and pig [62, 64–66]. Histological examination of the antral follicle of NPPC- and NPR2- mutant mice revealed that precocious meiosis resumption occurred in the oocyte with disorganized chromosomes or fragmented ooplasm immediately before ovulation [67]. Consistently, it was found recombinant mouse NPPC, but not NPPA or NPPB, stimulates the NPR2 expression in the cumulus granulosa cell and prevented spontaneous oocyte resumption through elevating cGMP levels in the cumulus granulosa cell and the oocyte during in vitro COC maturation [62]. This is in agreement with the finding that cGMP was generated by NPPC in the cumulus granulosa cells and diffused into the oocyte to inhibit meiotic resumption during in vitro pig COC maturation [68]. These observations suggest the maintenance of oocyte meiotic arrest requires the activity of NPPC/NPR2 signalling in the follicular somatic cells.

**Table 1:** The summary of NPR family within the follicle

Receptor	Coding Gene	Property	Oocyte	Mural Granulosa cell	Cumulus granulosa cell	deficiency
NPRA	NPR1	guanylyl cyclase receptors			Bovine [71]	protects C57BL/6 mice from ovarian cancers [107]
NPRB	NPR2	guanylyl cyclase receptors	Bovine [71, 74]	Mice [62]; Human [19]	Mice [62]; Pig [64, 108]; Bovine [71]; Human [19]; Cat [109]	female sterility [110]; Precocious meiosis resumption [111]
NPRC	NPR3	non-guanylyl cyclase receptor		Bovine [71]	Bovine [71]

**Table 2:** The summary of NPP family in the follicle

Ligand	Coding Gene	Receptor	Oocyte	Mural Granulosa cell	Cumulus granulosa cell	Others	deficiency
ANP	NPPA (Natriuretic Peptide Precursor A)	NPR1 NPR3	Pig [112];	Pig [112]; Rat [112]	Bovine [71]; Goat [113]	Pig follicular fluid [112]; Bovine corpora lutea [114]	
BNP	NPPB (Natriuretic Peptide Precursor B)	NPR1 NPR3		Goat [113]	Goat [113]		
CNP	NPPC (Natriuretic Peptide Precursor C)	NPR2 NPR3		Mice [62, 72]; Pig [64, 108]; Human [65]; Goat [113]; Cat [109].	Bovine [71]; Goat [113]	Human follicular fluid [65]	Precocious meiosis resumption [111]

Despite that NPPA was also detected in the mural granulosa cell, the oocyte and the follicular fluid in pig [69], given the fact that its functional receptor, NPR1 is undetectable within any type of the follicular cells yet, this review tends to believe it might play a different role compared with NPPC. Interestingly, although no report suggests NPPB is expressed by pig follicle, study shown the recombinant pig NPPB peptide, but not recombinant human or rabbit NPPB peptide, can maintain mouse oocyte meiotic arrest in a dose-dependent manner via upregulating cGMP production in the cumulus granulosa cell, additionally, this inhibition can be completely reversed by treatment with the NPR2 inhibitor [70]. These controversy phenotypes may be attributed to the sequence homology among the natriuretic peptides: the amino acid sequence of NPPA and NPPC is highly conserved among mammalian species. For example, the coding sequences of NPPC precursor mRNA are identical among human, mouse, rat, and pig [69]. In contrast, the NPPB sequences are different across those species [70]. However, it is currently unclear that how the ovary selective expresses different members of NPP and NPR family and its preference of NPPC/NPR2 cascade.

Unlike the unique expressed pattern of NPR2 limited in the granulosa cell of mouse and pig, the more recent study shows its mRNA was also detectable in the bovine oocyte, despite the other two NPR family members, NPR1 and NPR3 were uniquely expressed in the cumulus granulosa cells [71]. Furthermore, treatment of NPPC peptide only caused inhibitory effect in the COCs group, but not DOs group for the mouse and pig [64, 72], suggesting the target of NPPC in mouse and pig is the cumulus granulosa cells rather not the oocyte. In contrast, study in cow demonstrated that none of the NPP family member (NPPA, NPPB or NPPC), individually or in combination, inhibited the rate of meiosis resumption of COC in vitro, despite NPPA and NPPC induced a significant increase in concentrations of cGMP in cumulus cells and oocytes after 3 h of culture. More interestingly, treatment of NPPA or NPPC prevented the elevation of cAMP level within the oocyte after 6 h of culture and ultimately induced meiotic resumption instead [71]. Researchers believed that these contradictory results observed in mice, pig and cow are due to the monotocous species adopt the NPP/NPR system differently compared with the polytocous species [71]. However, whether the role of NPP/NPR in the bovine follicle stimulating oocyte meiotic resumption is still debatable as an opposite conclusion was derived by another lab, it was reported that NPPC can be used to delay bovine oocyte meiotic resumption and increase the oocyte developmental competence in vitro instead [73]. In addition, another study found NPR2 was detected in the bovine oocyte and it can be directly activated by mural granulosa cell-derived NPPC or estrogen [74]. Consistently, recently study found an overactive CNP/NPR2 was detected in anovulation ovaries from dehydroepiandrosterone (DHEA)-induced PCOS-like mouse, and it correlated with persistent high levels of estrogen [75].

Anyhow, although discrepancies were derived for bovine researches, it seems the mammals adopt a similar NNPC/NPR-cGMP-PDE3A signalling cascade system to responsible for generation of cGMP. However, it is still a big challenge for researchers to delineate the primary principle determinates the differential expression of NPPs/NPRs within the follicle cells.

## Oocyte carries the communication by regulating cGMP synthesis

Recent studies that the activity of inosine-5′-monophosphate (IMP) dehydrogenase (IMPDH, also termed guanosine monophosphate reductase [GMPR]) in the cumulus granulosa cells, and the influence of the oocyte on its expression collaboratively provide a new perspective for reproductive scientists to draw the bigger picture of oocyte meiosis arrest.

IMPDH is a purine biosynthetic rate-limiting enzyme in the biosynthesis of guanylyl metabolites by catalyzing IMP to xanthosine-5′-monophosphate (XMP) which is converted into GMP under the GMP synthase, then through a series of enzyme activity, the GMP turns into GTP, which services as guanylyl substrate for the guanylyl cyclise including NPR2 [76]. Previous study indicated that treatment of mizoribine or mycophenolic acid, two specific dehydrogenase inhibitors for IMPDH, induced a rapid precocious gonadotropin-independent resumption of adult mouse oocyte meiosis in vivo [77]. Similarly, treatment with these two inhibitors in the unmatured mice caused a premature oocyte meiotic maturation and resulted in a significant loss of developmental capacity [78]. However, during the past two decades, the researchers were puzzled by the underlying mechanism that IMPDH affects oocyte meiotic arrest. Until recently it was found that crucial role of IMPDH in maintaining meiotic arrest is through two coordinated pathways: [1] maintains oocyte meiotic arrest by catalysis of IMP to generate more substrate for NPPC/NPR2 system in the cumulus granulosa cell; [2] maintains the basal level of hypoxanthine (HX) in the follicular fluid which acts like an oocyte phosphodiesterase inhibitor to augment the meiotic arrest and intracellular cAMP accumulation [79].

Millimolar level of HX was detected in the follicular fluid in mouse [80, 81], and pig [82]. Although IMP can be produced either the *de novo* pathway or by salvage of HX, its main origin is rather *de novo* synthesis but not salvage of HX in the COC [83, 84]. Interestingly, the decrease of HX in follicular fluid can not induce the oocyte maturation under *in vivo* situation, However, the equivalent concentration is sufficient to maintain meiotic arrest by inhibiting cAMP-PDE activity to sustain elevated oocyte cAMP levels *in vitro*, and this meiotic arresting can be reversed by inhibition of IMPDH [85]. These finding suggest the meiotic arrest action caused by HX is rather independent of NPPC/NPR2 system, but both are under the control of IMPDH activity. For the future, it may be of interest to generate a homologous IMPDH knockout animal model and further confirmation of its role for the maintenance of meiotic arrest.

**Fig. 2:**
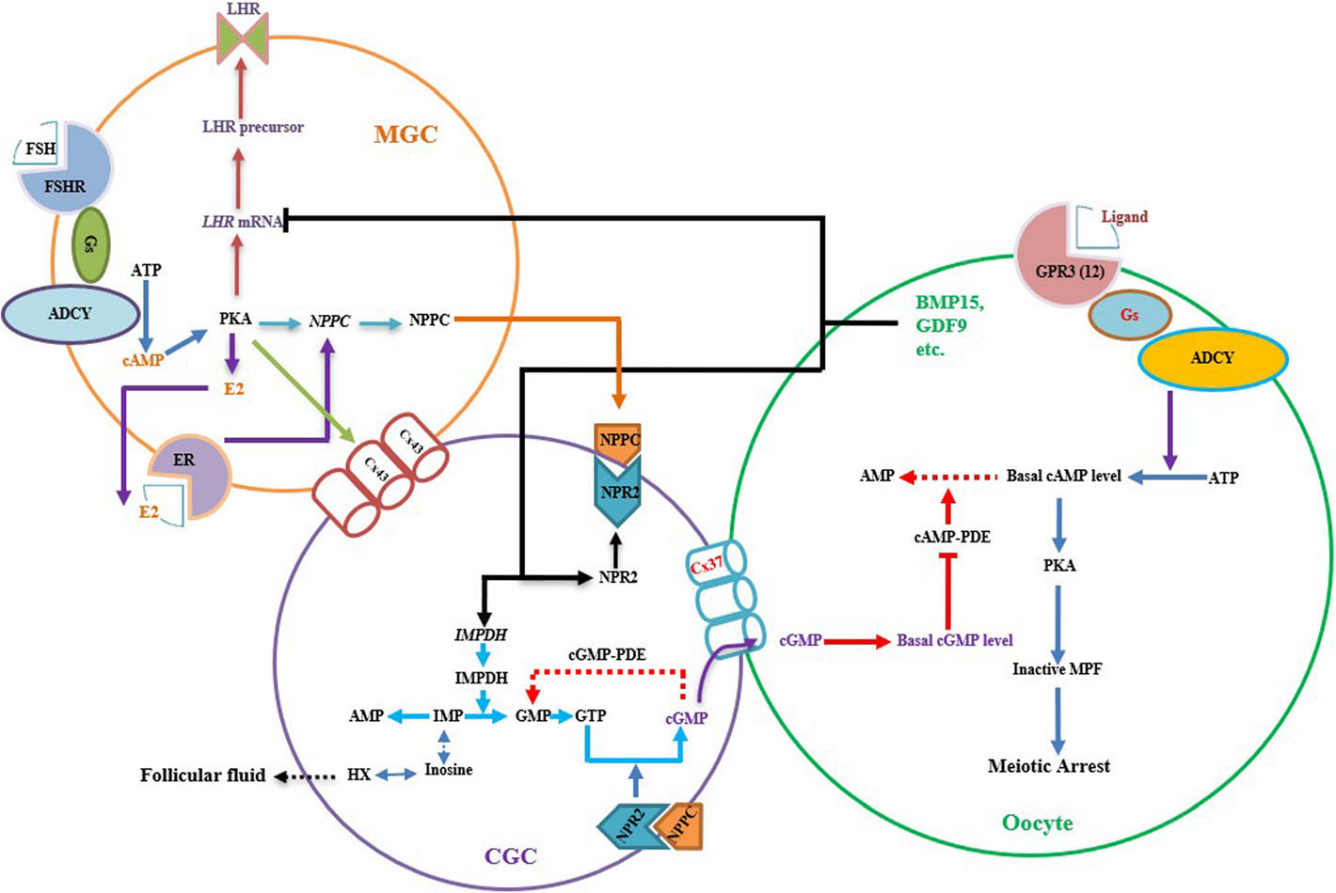
A abbreviated pathway depicting the participation of FSH/FHSR, Estrogen/ER, NPPC/NPR2, Oocyte in maintaining mammalian oocyte meiotic arrest. [1] In the mural granulosa cells, FSH binds its GPR receptor (FSHR), collaborating with Estrogen/ER signal pathway prompts NPPC production [72, 91, 103]. [2] In the cumulus granulosa cell, NPPC actives its receptor NPR2, converts GTP into cGMP, then cGMP diffuses into the oocyte through gap junctions (mainly Cx37) inhibits cAMP-PDE activity, blocks the degradation of intraoocyte cAMP [84, 104]. [3] The oocyte-derived paracrine factors increase cumulus cell NPR2 expression leads to an elevated cGMP level in both cumulus granulosa cell and oocyte; In addition, IMPDH is increased, converts IMP to GMP, to provide more substrates required to produce guanylyl metabolites and cGMP in the cumulus granulosa cell; Furthermore, the increased IMPDH maintains the basal level of HX in the follicular fluid, which might act as an oocyte phosphodiesterase inhibitor to augment the meiotic arrest and intracellular cAMP accumulation [95, 105]. ER: G protein-coupled estrogen receptor; IMPDH: inosine monophosphate dehydrogenase; IMP: inosine-5′-monophosphate; HX: hypoxanthine

To explore the possibility of the oocyte in the regulation of NPPC/NPR2 system and IMPDH, the oocyte was removed from mouse and pig COCs (Oocytectomy, OOX), a significantly reduced expression of *NPR2* mRNA in the cumulus granulosa cells was found; While, Co-cultured these cumulus granulosa cell with full grown DO, or treatment with two of the oocyte-derived paracrine factors (BMP15, GDF9 and FGF8B) completely restored *NPR2* mRNA to that observed in intact complexes [62, 64]. In addition, significant decrease in the expression of two IMPDH family members, *IMPDH1* and *IMPDH2* mRNA levels in the mice cumulus granulosa cells was observed when the oocyte was removed, in contrast, co-cultured these cumulus granulosa cell with GV stage DO reversed their mRNAs level as same as of intact COCs [62]. These observations suggest the maintenance of meiotic arrest caused by NPPC/NPR2 and IMPDH is still under the manipulation of the oocyte itself (Fig. 2). However, it is currently unclear whether this regulation is direct. Some oocyte-derived paracrine factors and signalling pathways may play important role during this process, the mechanism under this regulation warrant further research.

## LHR signalling decreases cGMP by modulating the NPPC/NPR system and the kinetics of the cGMP decrease within the follicle

It’s well known that, in response to a preovulatory surge of LH from the pituitary gland during each reproductive cycle in mammals, oocytes initiate the meiotic resumption from the first meiotic prophase arrest [11]. To date, it is well accepted that the underlying mechanism is because the LH causes significant decrease of cGMP in the surrounding granulosa cells, in turns decreases the cGMP level within the oocyte and releases the cAMP-PDE activity, subsequently leading to degradation of cAMP in the oocyte, and the decrease of cAMP ultimately activates oocyte MPF for the successful resumption of the meiosis [61, 86]. However, the dynamic change and transfer of signalling caused by LH surge occur rapidly and precisely within the follicle, the approaches used in the studies of oocyte meiotic arrest and resumption are limited by their accuracy and sensitivity in monitoring cGMP dynamics in living cells. In the past a few years, the combination of fluorescence resonance energy transfer (FRET) technique with cyclic nucleotide sensors to visualize the intercellular trafficking of cGMP in real-time within the live follicle has facilitated research in the topic greatly [87], revealing the kinetics of some key events happen after LH surge within in the mouse follicles [60, 83, 84]. Here, this review will summarize the major findings obtained previously in the term of the mechanism that activated LH/LHR signaling reinitiate meiotic resumption, furthermore, to provide suggestions for future research focus.

Within the ovary, the LHR signaling needs to travel multiple cell layers to the oocyte, because the main targets of LH are the theca cells and mural granulosa cells [88]. The theca cells provide structural support for the growing follicle and synthesize androgen in response to LH, which is transported into neighboring mural granulosa cell to act as the substrate of aromatase [89]. However, the finding that the cGMP level remained at a constant low level and no change in the theca cells before and after LH exposure suggests that theca cells may not be involved in resuming meiosis within the mouse follicle. The absence of the theca in the regulation of meiotic resumption is probably due to the lack of gap junction between the theca cells and the granulosa cells in which the cGMP flow usually transfers through one cell to another [120, 132]. Therefore, it’s reasonable to suspect the granulosa cell but not the theca cell is involved in the down-regulation of cGMP after the preovulatory LH surge. During the past few years, some evidences have been derived from both in vivo and in vitro studies.

**Fig. 3:**
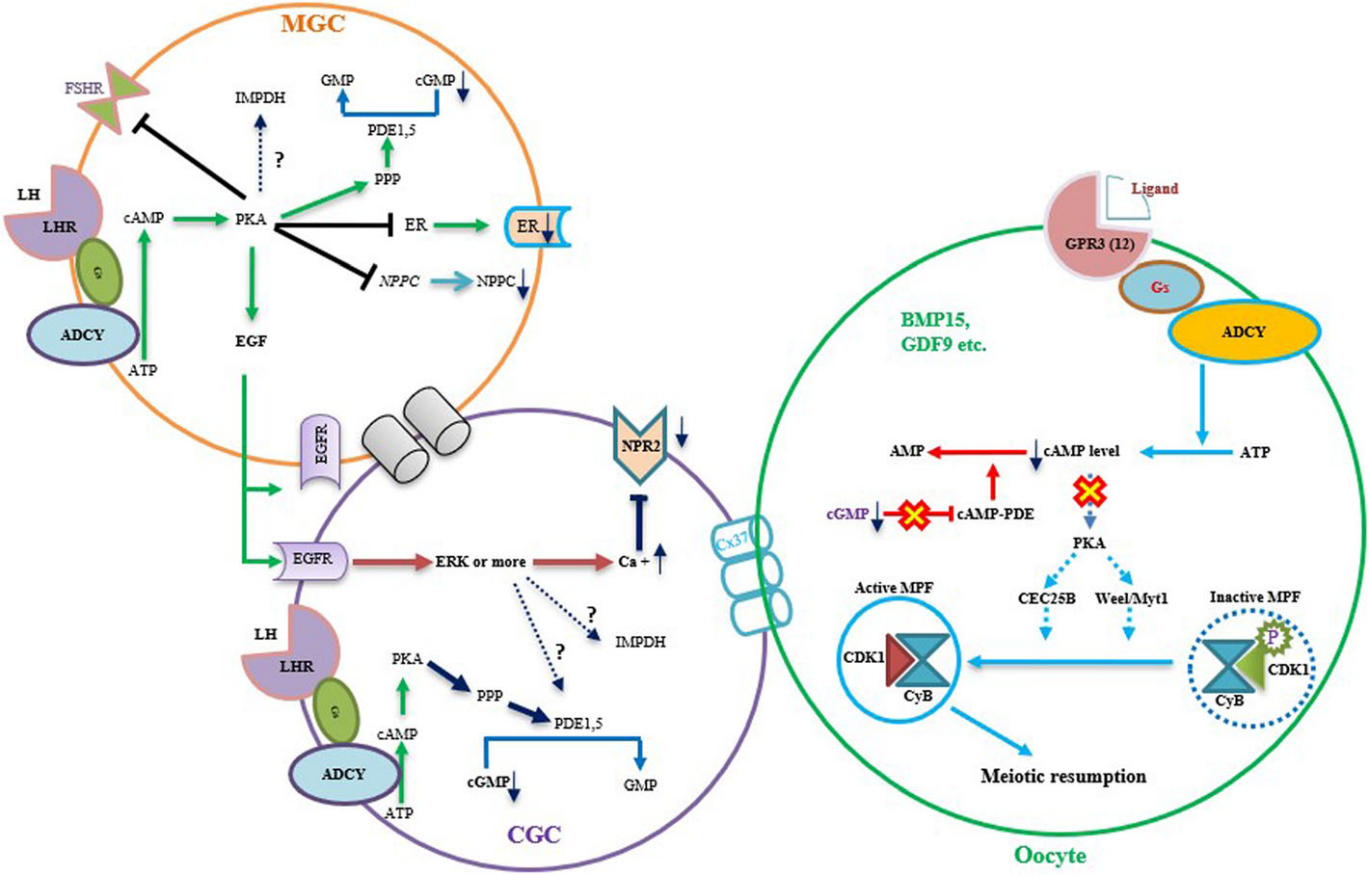
Schematic model depicting the proposed signaling pathway in LH/hCG-induced the resumption of meiosis. LH surge results in the dephosphorylation and deactivation of NPR2 via the phosphoprotein of phosphatase (PPP)-family in the cumulus granulosa cell [96], NPPC production and secretion in the mural granulosa cell. The LH/LHR-induced activation of cAMP/PKA increases the following compounds in the mural granulosa cell: phosphorylation of the cGMP-PDE5, EGF and EGF-like factors production and secretion, and ER expression [103]. The rapid phosphorylation of the cGMP-PDE5 is responsible for converting cGMP into 5’GMP, causes the outward diffusion of intraoocyte cGMP, leads to the release of cAMP-PDE activity within the oocyte, in turn, the activated cAMP-PDE catalyses the cAMP into AMP, ultimately results in meiotic resumption [99]; The production of EGF and EGF-like factors activates the EGFR signaling elevates calcium of cumulus granulosa cells further to inactivate NPR2 [106]; Furthermore, the decrease of ER serves as an augment step [103]. cGMP-PDE5: cGMP phosphodiesterase 5; EGF-like factors: amphiregulin (AREG), epiregulin (EREG), and β-cellulin (BTC)

From in vivo studies, ELISA assay indicated that the ovarian NPPC decreased starting at 2 h after hCG injection, and this is corresponding to the time the oocyte nuclear envelope starts to breakdown, indicating its decrease potentially contribute to stimulating GVBD [90]. Consistently, it was observed that the decreased NPPC level within the ovarian follicular fluid of In Vitro Fertilization (IVF)-embryo transfer or Intracytoplasmic Sperm Injection (ICSI)- woman after treatment with the similar ovulatory dose of LH/hCG [65]. Interestingly, compared to the LH signaling causes the NPPC decrease at 2 h, it was found within 20 min pre-ovulatory LH/human chorionic gonadotropin (hCG) stimulation caused a rapid decreased NPR2 guanylyl cyclase activity without a corresponding decrease in NPR2 protein in the mouse mural granulosa cell [90]. By using FRET sensor to visualize the real-time intercellular cGMP flow in the live mouse follicles indicated the cGMP in the mural granulosa cell, cumulus granulosa cell and oocyte have decreased to the uniform low level after 20 min of LH stimulation [60, 83], despite the decreased NPR2 activity in the cumulus granulosa cells occurred only after 2–3 h [90]. Suggesting LH-induced NPR2 activity decrease in the mural granulosa cell is the prerequisite for the initiation of meiosis; Whereas, the decreased NPR2 activity in the cumulus granulosa cell might not contribute to initiation of cGMP downregulation in the response to the LH surge. This review tends to believe it might function as an augment step to maintain the continuous low cGMP level for the success and completion of meiotic resumption (Fig. 3).

Interestingly, in vitro studies indicated that EGF but not LH caused a decline of *NPPC* mRNA and cGMP level in the cultured mouse mural granulosa cells [91]; In addition, treatment with EGF during mouse COC culture decreased NPR2 transcript and its guanylyl cyclase activity in the cumulus granulosa cells and reversed NPPC-induced meiosis arrest in the oocyte, despite the decrease of *NPR2* mRNA is believed not contribute to the meiotic resumption [91, 92]. Given the well-accepted fact that LH-induced intrafollicular EGF-like family are indispensable for oocyte re-entry into the meiotic cell cycle and other critical physiological processes [93, 94], we believe that the observed NPPC decrease in the granulosa cell and follicular fluid after LH/hCG admiration in mammal is, at least partly, through the action of LH signaling-derived EGF like factors. The EGF or EGF-like factors in the mural granulosa cells act as autocrine factors to binds to its membrane EGF receptor (EGFR) to cause the decrease of NPPC; On the other side, these mural granulosa cell derived EGF-like factors might function as paracrine factors to induce the decrease of NPR2 expression in the cumulus granulosa cell. Thus, the effects of these EGF-like factors on both the mural and cumulus granulosa cell are collectively to further block of cGMP generation and contribute to the meiosis resumption, and act as an augment step to the predominant LH-induced NPR2 guanylyl cyclase activity in the cumulus granulosa cells [92] (Fig. 3).

More interestingly, recent study demonstrated the intrafollicular flow of cGMP is dual directions: [1] The granulosa cell-derived cGMP travels through gap junctions from the surrounding granulosa cell to the oocyte is responsible for maintaining meiotic arrest at dictyate prophase I before the LH surge; [2] In the response to preovulatory LH surge, cGMP concentration decreases in a sequential order from the mural granulosa cell, the cumulus granulosa cell, and the oocyte, the outward diffusion cGMP ultimately prompts the oocyte to reinitiate meiosis [95, 96]. Although number previous studies demonstrated the closure of gap junction is required for the decrease of intrafollicular cGMP and subsequent meiosis resumption [126–128], the finding that no decrease in the gap junction permeability was observed before the cGMP level throughout of follicle has decreased to a uniformly low level. Suggesting the rapid outward diffusion of cGMP caused by LH within follicle occurs prior to the LH-induced decrease in the gap junction permeability. Whereas, the LH indeed induces the phosphorylation of the main gap junction component Cx43 and results in the closure of gap junction between the somatic cells, prior to GVBD [97], this review believes the following closure of gap junction after the rapid decreased cGMP acting as a augment step to further guaranty an low level of cGMP within the oocyte or cumulus granulosa cells.

However, it’s believed that only part of cGMP decrease in the granulosa cell is mediated by the rapid dephosphorylation and inactivation the NPR2 guanylyl cyclase in response to LH, and the mechanism for the remainder of the LH-induced cGMP decrease remains unexplained [98]. Studies demonstrated that phosphorylation and the increase in activity of some cGMP-Phosphodiesterase (cGMP-PDE) family members, specifically the cGMP-PDE5, contributed to the LH-induced resumption of meiosis in the rodents [99, 100]. The blocking of LH-induced PDE5 phosphorylation by the mutation of PDE5 serine 92 to alanine (PDE5-S92A) had no effect on the timing of meiotic resumption, suggesting multiple PDE family members must be activated by LH signaling to account for the rest of the cGMP decrease [100]. Furthermore, the selective expression of PDE family varies in different species. For example, only little or no PDE6A, PDE6B, or PDE6C protein were detected in rat follicles, in contrast, it was found PDE6C significantly increased in the porcine COC during in vitro maturation [101]; In addition, PDE6C, PDE8A and PDE11A have shown immunostaining positive in porcine mural granulosa cell membranes and the cytosol [102].

## Summary and prospective

In the mammalian oocyte, the elevated level of cAMP is required to maintain meiotic arrest at *dictyate prophase I*. The oocyte itself adopts a unique PGR-Gs-NDCY system to generate sufficient endogenous cAMP in cooperation with the inhibition of PDE3 activity. Although the oocyte-derived cAMP is essential for maintaining meiotic arrest, cGMP generated by the surrounding granulosa cells is required for maintaining elevated cAMP level via its suppression of PDE3 activity in the oocyte. The mural granulosa cell and the cumulus granulosa cell express NPPC and its receptor NPR2, respectively; The selective expression and activation of NPPC/NPR2 system is collaboratively responsible for producing cGMP within the follicular granulosa cells. In addition, the IMPDH converts IMP to GMP to prepare more substrates for NPR2 activity to sever as a complementary step to further warranty the elevated intraoocyte cGMP. However, the activity of NPP/NPR system and IMPDH action are monitored by the oocyte itself via secreting paracrine growth factors include GDF9 and BMP15. Hence the oocyte orchestrates the synthesis of cGMP in the surrounding granulosa cells, collaborating with its own cAMP producing via GPR-Gs-ADCY cascade, precisely maintain meiotic arresting at prophase of meiosis I before the LH surge.

When the LH surge occurs, as the very first step, LH signaling induces the dephosphorylation and inactivation of the NPR2 guanylyl cyclase, results to a rapid drop of cGMP concentration in the granulosa cell and the oocyte in a sequential order via gap junctions. The outward diffusion of cGMP occurs prior to the LH-induced closure of gap junction might attribute to the following two reasons: [1] The reduced gap junction permeability might act as an augment step to further enhance the low concentration of cGMP in the oocyte; [2] The rapid decrease intraoocyte cGMP cause the release of the oocyte-derived factors that suppress some key genes including NPR2 and LHR in the granulosa cell, thus enhances the maximum effort of LH stimulation. On the other hand, to supplement with this cascade, the induction of the EGF-like growth factors in the granulosa cell caused by LH might act as paracrine or autocrine mediators to trigger the EGFR pathway further to decrease the expression of NPPC and NPR2. Overall, these signaling pathways coordinately propagate the LH effect throughout the follicle and ensure the persistence of meiotic progress.
